# Annotations

**Published:** 1907-01-19

**Authors:** 


### ANNOTATIONS.

### The Indigestibility of Plummer's Pill.

The necessity for constant attention to the niceties of pharmaceutical combination in the prescribing of drugs, is well illustrated by the communications on the subject of Plummer's pill recently addressed to the medical and pharmaceutical Press by Sir James Sawyer. In treating a patient with some pills made according to the official formula Sir James noticed that no physiological effects were produced, and this led to an examination of the stools and to a discovery of the fact that the pills had passed through the alimentary canal without change. Further tests showed that the pharmacopœial formula yielded pills which could be digested in water for thirty-six hours at a temperature of 100° F. without being affected. It is suggested that this is due to a combination between the resin of guaiacum and the alcohol, the latter being one of the excipients ordered in the Pharmacopœia. In this way it is believed there is formed a resinous mass which causes the rest of the ingredients to "set" into so firm a substance as to resist the action of the gastro-intestinal fluids. Important as this is in itself, it becomes of still more importance in view of the proposal to omit in future the castor oil now used in making up the pill, and to use alcohol as the sole excipient. Sir James Sawyer has referred to Dr. Plummer's original formula and finds the excipient (if so it may be called) there ordered to have been "balsamum capivi." Even this, however, does not yield a pill which readily undergoes disintegration. After various trials Sir James has come to the conclusion that the best excipient is "syr. glucosi," and he accordingly advises the following formula: Calomel, sulphurated antimony, of each 1 grain, guaiacum resin, 2 grains, syrup of glucose, a sufficiency.

### Intestinal Parasites and Pernicious Anæmia.

The possible mischief which may result from the presence of parasites in the human alimentary canal has always been a difficult clinical problem. On the one hand is the undoubted fact that in numbers of instances in which such parasites are present the health of the host appears in no way disturbed; on the other, it is certain that in many cases serious and even alarming symptoms have promptly subsided on expulsion of some variety of parasite. For the most part, such symptoms, when they have existed, have been attributed to the directly irritating effects of the parasite upon the nervous structures of the intestine, there being produced in this way central or reflex nervous disturbances. More recently it has been suggested that in place of, or in addition to, this mechanical irritation, an intestinal parasite may prove harmful to its host as a result of the absorption of some secretion manufactured by the parasite. It has even been held that the anæmia associated with such a parasite as the ankylostoma duodenale is to be explained in this fashion, rather than as a result of loss of blood following the damage inflicted by the worm on the intestinal mucous membrane. Dr. Bilderbeck Gomess has now somewhat modified this position. He accepts the view that ankylostoma may be directly responsible for severe anæmia and even for a variety of pernicious anæmia. Bothriocephalus latus, which has been accused on a similar indictment, he discharges with a verdict of "not proven." At least he does this so long as the parasite is healthy. But if in its turn the bothriocephalus is the victim of parasitism, that is, if it is affected with gregarines, another state of matters arises. Further, the ascaris lumbricoides may become infected in similar fashion, and then, equally with the bothriocephalus, may be associated with pernicious anæmia. The existence of gregarines in man has been generally denied, but Dr. Gomess combats this statement. He affirms, on the contrary, that they occur in considerable numbers when associated with ascaris lumbricoides, that is to say, when the ascaris is itself infected with gregarines.

### The Cure of "Sleeping Sickness."

Professor Koch, who has recently turned his attention to sleeping sickness, has published a series of reports on the subject, his last one putting forward the claim that "atoxyl," an arsenious preparation, will probably prove as efficacious a specific in the case of sleeping sickness as quinine is in that of malaria. Before entering into a discussion on this statement, it is as well to state that the use of "atoxyl" in trypanosoma infections is not new. The Liverpool School of Tropical Medicine have been using this drug in the treatment of animals infected with trypanosomes for a considerable time, and have published their results, which on the whole are satisfactory. If, then, the drug proves to do good in human cases the merits of the discovery do not go to Koch, but to the school already mentioned. As to the statement that it will prove as efficacious as quinine is in malaria, one must reserve one's opinion until at least years have elapsed. Koch, in his report, admits this to a certain extent, but the times he gives are manifestly too short for any degree of certainty to be obtained. For example, he writes: "In two or three months, according to circumstances, we hope to see the complete cure of the majority of the patients. We must then keep them under observation for an equal length of time to see if relapses ensue. It will only be when we are sure that the cure endures after the administration of atoxyl has ceased, that we shall be able to regard our problem as solved. That the prevention of the disease will follow the cure of the sufferer is obvious." To anyone who has studied sleeping sickness closely, this dictum of Koch's is at once seen to be far too optimistic, much of the same type, in fact, as his idea of stamping out malaria by the universal giving of quinine. That arsenic and arsenical preparations do good is undoubted, and this is of course an advance; but European cases heavily drugged by arsenic, though having their lives prolonged, have eventually succumbed to their malady, in some cases years afterwards. With this before us, then, it is well to receive Koch's statements with caution, and to withhold an opinion till the necessary time for testing it has elapsed.

